# The use of the Brief Child Abuse Potential Inventory in the general population in Finland

**DOI:** 10.1080/02813432.2019.1571002

**Published:** 2019-01-28

**Authors:** Noora Ellonen, Heidi Rantanen, Sari Lepistö, Mika Helminen, Eija Paavilainen

**Affiliations:** a Institute of Criminology and Legal Policy, Faculty of Social Sciences, University of Helsinki, Helsinki, Finland;; b Faculty of Social Sciences, Tampere University, Tampere, Finland;; c Tampere University Hospital, Tampere, Finland;; d Southern Ostrobothnia Hospital District, Seinäjoki, Finland

**Keywords:** Child abuse, child maltreatment, children, family practice, Finland, parents, prevention, risk

## Abstract

**Objective:** The purpose of this research was to analyze psychometric information in the Brief Child Abuse Potential Inventory (BCAP) in a Finnish general population sample.

**Design:** A self-report survey of parents in a primary health care setting and a hospital setting was used to evaluate the use of the BCAP.

**Setting:** The study population consisted of parents who were visiting one of the following contexts: a primary maternity health care clinic, a child health care clinic, and the maternity outpatient clinic, various pediatric outpatient clinics, the general pediatric ward, the pediatric surgical ward, or the neonatal intensive care unit in a hospital setting.

**Subjects:** The BCAP was given to parents at the 30–34th week of pregnancy, when the child was 5 months old or all parents depending on the context. The BCAP was delivered to 759 parents. The final size of the sample was 453 respondents.

**Main outcome measure:** The BCAP, which consisted of 25 items to screen child abuse potential and nine items for evaluation of respondent validity.

**Results:** The internal consistency of the Abuse Risk Scale was good (.770), and the validity scales worked well. The factor structure mirrors with the original factors structure.

**Conclusion:** The psychometric properties of the BCAP reported in the analysis suggest that the BCAP could be a valid instrument to detect child abuse potential in the general population in Finnish health care settings. However, among Finnish respondents there is very little variation in some parts of the measure, which suggests that further research should assess the validity of the instrument in representative samples. Further analysis is also needed to evaluate the correct classification rate of the BCAP.

**Key points**
Identification of families at risk of child maltreatment requires valid tools to recognize risk within the general population, as part of child and family needs and risk assessments in family services.
1. The BCAP is valid, reliable, and useful in bringing parental worries under discussion in child and family services.
2. Results of this study can be used for a more systematic and valid child maltreatment risk assessment for identifying families who need help managing their everyday lives.

## Introduction

It is a commonly shared understanding that children should not be exposed to any type of maltreatment. Nevertheless, children experience it also in Nordic countries, although all kind of child maltreatment have been banned for decades [1]. Based on a Finnish self-report survey in 2013, three per cent of 9th graders reported being slapped by their parent during past 12 months, and 21% reported experiences of mild physical violence by their parents at any point in their lives [2]. Based on self-report survey conducted to parents of children under 12 years, four per cent of Finnish parents and three per cent of Swedish parents report having slapped to their child [3]. Compared to other countries, such as Canada [4] or England [5] these rates are significantly lower.

Research has suggested several risk factors for child maltreatment, which can be divided into child-, parent-and family-related risk factors. Known child-related risk factors are, for example, child’s disability, behavioral problems or irritability of the child. Risk factors related to the parents may include substance abuse, mental health problems, emotional immaturity, lack of social support, parental history of maltreatment as a child and unrealistic expectations regarding the child. Known family-related risk factors are, for example, several children in the family, low income, socioeconomic disadvantage, history of child protective interventions and family perception of lack of social support. The accumulation of any kind of risk factors also increases the risk of child maltreatment as well as any kind of maltreatment taking place is a known indicator for further maltreatment [6]. Negative consequences of child maltreatment are also evident. Maltreatment is associated with mental problems, behavioral problems, substance abuse, obesity and some chronic deceases, increased risk of suicidal behavior and risky sexual behavior [7].

Various measures exist to help professionals assess parenting competence and screen for child maltreatment [8,9]. One of the most frequently used internationally and validated measures for detecting potential child maltreatment is the Child Abuse Potential Inventory (CAP) created by Joel Milner in 1986 [10]. The CAP Inventory is a self-report measure of 160 forced-choice (agree/disagree) questions. Of the 160 items, 77 constitute an Abuse Risk Scale consisting of six sub-scales: Distress, Rigidity, Unhappiness, Problems with Child and Self, Problems with Family, and Problems with Others. The CAP Inventory includes three validity scales and three validity indexes to evaluate the reliability of the responses: The Lie Scale, the Random Response Scale, the Inconsistency Scale, the Faking-good Index, the Faking-bad Index, and the Random-response Index [10].

High internal consistency and test-rest reliability of the original CAP Inventory have been reported. The correct classification rate of known abusers and non-abusers ranges from 80% to 90% [10–12]. It has good psychometric properties and substantial literature supporting the measure’s validity.

The CAP Inventory has been translated into multiple languages, and it has been used around the world. The translated versions of the Abuse Risk Scale have also been proven to be reliable and valid. Internal consistency of the Abuse Risk Scale varied from .88 to .91 (Cronbach’s alpha), and correct classification rates based on discriminant analyses varied from 83.0% to 100% [13]. The only significant differences have been reported in the factor structure of the CAP Inventory and in the Lie Scale of the validity section [11,13]. The CAP Inventory has also been validated in the Finnish general population, and the scale has shown to be a valid measure in that context [13,14].

Despite the advantages of the CAP Inventory, some characteristics, such as the length of the questionnaire and the complex scoring system, limit the measure’s usefulness, especially in some primary health care settings. Because of these limitations, Ondersma et al. [15] developed a brief version of the measure. The brief version (BCAP) includes 24/25 items and 9 validity items of the original CAP. Items were selected to shorten the CAP but to retain as much shared variance with the full measure as possible, to retain a stable factor structure and a useful validity scale, and to maximize the BCAP’s predictive validity [15]. Based on the American sample, the BCAP has seven sub-scales: Distress, Family Conflict, Rigidity, Happiness, Feelings or Persecution, Loneliness, and Financial Insecurity. As validity scales, the BCAP includes the Lie Scale and the Random Response Scale. Psychometric properties of the BCAP have been reported to be good, and the BCAP overlaps extensively with the full CAP Abuse Risk Score [15].

The BCAP has not yet been widely used internationally and is not well validated in general populations. The BCAP has been used in Japan in a general population sample but not validated in it [16]. The only evaluation of the use of the BCAP among a general population was performed in the United Kingdom (UK) [17]. In that analysis, the overall reliability of the Abuse Risk Scale was good (Cronbach’s alpha .816), and the factor structure was almost the same as in the US sample [15]. Only minor culture-related differences were found. However, from the UK perspective, the validity scales (Lie Scale and Random Response) need development. But the analysis suggested that the BCAP may be a reliable, quick, and useful tool for clinical screening for increased risk of physical child abuse in parents in the UK setting [17].

The purpose of this research is to analyze psychometric information in the BCAP in a Finnish general population sample. In addition, the factor structure and the suitability of the validity scales are tested. By doing this, the aim is to find whether the BCAP is useful in assessing child maltreatment, described as parental worries by parents themselves, within the general population.

## Material and methods

### Study design

To evaluate the use of the BCAP in the Finnish general population. The BCAP was delivered as a one-occasion, self-report survey to parents in a primary health care setting and a hospital setting between January 2017 and March 2018.

### Study population and process

The BCAP was delivered to 759 parents of whom 464 returned questionnaires, resulting response rate of 61. There are no data available of those, who didn’t response, so the representativeness of the data can’t be evaluated. From the reliability point of view, we applied the rule used in the CAP Inventory that if the respondent has more than 10% missing responses, the questionnaire should be considered invalid. Therefore, 11 respondents who had at least three missing responses were excluded from further analysis. The final size of the sample was 453 respondents.

The study population consisted of parents who visited one of the following contexts: a primary maternity health care clinic (54 received responses), a child health care clinic (38), and the maternity outpatient clinic (76), various pediatric outpatient clinics (53), the general pediatric ward (119), the pediatric surgical ward (54), or the neonatal intensive care unit (25) of which the first two belong to primary health care and rest to specialized care. The BCAP was given to parents at the 30–34th week of the mother’s pregnancy, when the child was 5 months old, or all parents depending on the context. Parents were not informed of the study before entering to the context. The BCAP was filled out at home before the next appointment or during the visit. Parents were given the possibility to fill out the form with the other parent, separately, or alone.

The majority of the respondents were female (82.3%, *n* = 373), and the mean age was 33.1 years (*SD* = 6.9). Most of the respondents had 1–2 children (56.1%), 31.8% had 3 or more children, and four per cent were expecting their first-born (missing 8.4%).

### Measure

The BCAP, renamed the Family Welfare Questionnaire, included a cover letter. A standardized back-translation procedure was performed previously with the CAP [18] in Finland and used in the BCAP. It consisted of 25 items as shown in Table 1. To enable quick checking of the BCAP in busy health care contexts, the Lie and Random Response scale items (shown in Table 2) were placed at the end of the questionnaire. Demographic questions consisted of the parent’s sex, age, number of children, and age of the children.

**Table 1.** The BCAP items and frequencies of items, % (*n*).

Items	Disagree	Agree	Missing
I am a happy person	0.0 (0)	100 (453)	0.0 (0)
Sometimes I feel all alone in the world	92.7 (420)	7.1 (32)	0.2 (1)
Everything in a home should always be in its place	81.2 (368)	18.1 (82)	0.7 (3)
I often feel lonely inside	89.8 (407)	9.3 (42)	0.9 (4)
Children should never disobey	95.8 (434)	3.1 (14)	1.1 (5)
I sometimes worry that I will not have enough to eat	98.2 (445)	1.8 (8)	0.0 (0)
People have caused me a lot of pain	94.0 (426)	5.7 (26)	0.2 (1)
My life is happy	0.4 (2)	98.9 (448)	0.7 (3)
Children should be quiet and listen	90.3 (409)	6.6 (30)	3.1 (14)
My family fights a lot	97.8 (443)	2.2 (10)	0.0 (0)
My family has problems getting along	96.7 (438)	2.6 (12)	0.7 (3)
I often feel worthless	96.9 (439)	2.9 (13)	0.2 (1)
Other people have made my life unhappy	96.2 (436)	3.5 (16)	0.2 (1)
I often feel very upset	90.9 (412)	8.6 (39)	0.4 (2)
I have a happy life	0.7 (3)	99.3 (450)	0 (0.0)
I am easily upset by my problems	90.9 (412)	8.2 (37)	0.9 (4)
I am often depressed	97.8 (443)	2.0 (9)	0.2 (1)
I am often upset	98.2 (445)	1.5 (7)	0.2 (1)
A child needs very strict rules	97.6 (442)	1.8 (8)	0.7 (3)
I am often upset and don’t know why	99.3 (450)	0.7 (3)	0 (0.0)
I often feel very alone	98.0 (444)	1.8 (8)	0.2 (1)
I often feel alone	90.7 (411)	8.8 (40)	0.4 (2)
My family has many problems	95.6 (433)	3.8 (17)	0.7 (3)
Other people have made my life hard	95.8 (434)	4.0 (18)	0.2 (1)
I sometimes worry that my needs will not be met	83.9 (380)	15.9 (72)	0.2 (1)

**Table 2.** The BCAP validity scale items and frequencies of the validity scale items.

Items	Disagree	Agree	Missing
I sometimes act without thinking	55.6 (252)	42.2 (191)	2.2 (10)
I know what is the right and wrong way to act	4.9 (22)	93.8 (425)	1.3 (6)
I sometimes lose my temper	30.0 (136)	68.2 (309)	1.8 (8)
It is okay to let a child stay in dirty diapers for a while	87.6 (397)	8.4 (38)	4.0 (18)
Sometimes I have bad thoughts	88.7 (402)	9.7 (44)	1.5 (7)
Children should not learn how to swim	99.6 (451)	0 (0.0)	0.4 (2)
I sometimes fail to keep all of my promises	38.0 (172)	60.9 (276)	1.1 (5)
People sometimes take advantage of me	81.7. (370)	16.6 (75)	1.8 (8)
I sometimes say bad words	29.4 (133)	68.2 (309)	2.4 (11)

### Statistical analysis

Cronbach’s alpha reliability coefficients were used to describe the internal consistency of the Abuse Risk Scale. Principal component analysis was used to find the suitable factor structure for this Finnish general sample. SPSS statistical software was used for the analysis.

## Results

### Frequencies

In Table 1, the frequencies of each item are presented. The table shows that in most items the number of missing answers is low. There is also very little variation in the responses to four of the items. Those items are: I am a happy person, my life is happy, my life is good, and I am often upset and do not know why. The lack of variation means that those items cannot be used in adjusting the factor structure. Therefore, those items were removed from the principal component analysis.

### Reliability and Finnish norms/cutoff points

The internal reliability of the measure (25 items) is good. Cronbach’s alpha is .770. If the four items with too little variation are removed, Cronbach’s alpha is .781. Finnish norms for the BCAP (mean scores and standard deviations) were calculated for the Abuse Risk Scale. The mean of the abuse score was 1.14 (*SD* = 1.20). In the UK sample (also from the general population), the mean was 5.89 (*SD* = 3.60), and in the four US samples, the means varied between 8.4 (*SD* = 6.4) and 9.4 (*SD* = 5.9).

In the BCAP, 9 or 12 have been used as a cutoff point for the Abuse Risk Scale. These numbers have been reported to present well of the cutoff scores (166 or 215) used in the long CAP Inventory [15]. Because the norms of the Abuse Risk Scale are significantly lower in the Finnish sample, the use is not reasonable. It would include only the top 0.4% of the distribution of the Abuse Risk Scale. The manual of the CAP Inventory also encourages the determination of locally appropriate cutoff points [10].

In the long CAP Inventory, a cutoff point of 100 was used in the Finnish setting, which is almost 20% of the maximum score (486). Based on the same reasoning, the appropriate cutoff point for the BCAP would be five. According to that cutoff point, 6% of the respondents have an elevated risk of child maltreatment.

### Validity of responses

The Lie Scale includes six items and the Random Response Scale three items. The frequencies of the items are presented in Table 2. Ondersma et al. [15] suggested that any score of four or above on the Lie Scale and one or above on the Random Response Scale should be considered invalid especially when considered together. According to these cutoff points, 66.7% of the responses would be valid according to the Lie Scale and 86.5% according to the Random Response Scale. When the scales are considered together, meaning that both scale scores are evaluated, only 5.9% of the responses would be invalid.

### Factor structure

All 21 items were subjected to a principal component analysis with promax rotation to analyze the factor structure. In the Finnish sample, a five-factor structure was the most suitable when eigenvalues, a contribution provided by the factors to the overall explained variance, and interpretability were considered. Loadings over .40 were selected for the factors (Table 3). Those factors are: Loneliness and distress, Impact of others, Family conflict, Rigidity and Financial insecurity.

**Table 3.** Factor structure of the items.

	Loneliness and distress	Impact of others	Family conflict	Rigidity	Financial insecurity
I often feel alone	.785				
Sometimes I feel all alone in the world	.714				
I often feel lonely inside	.692				
I am often depressed	.621	.517			
I often feel very alone	.613				
I often feel very upset	.583				
I often feel worthless	.563	.623			
I am often upset	.415				
I am easily upset by my problems	.406				
People have caused me a lot of pain		.787			
Other people have made my life hard		.76			
Other people have made my life unhappy		.745			
My family has many problems		.718			
My family has problems getting along			.794		
My family fights a lot			.78		
I sometimes worry that my needs will not be met			.608		
Children should be quiet and listen				.774	
Children should never disobey				.696	
Everything in a home should always be in its place				.576	
I sometimes worry that I will not have enough to eat					.627
A child needs very strict rules					–.517

## Discussion

### Principal findings

In this Finnish general population sample, the internal consistency of the Abuse Risk Scale was good (Cronbach’s Alpha .770).The mean BCAP Abuse Score (*M* = 1.14, *SD* = 1.20) was considerably lower than in any other studies applying the measure. In the UK sample (also from the general population), the mean was 5.89 (*SD* = 3.60), and in the four US samples, the means varied between 8.4 (*SD* = 6.4) and 9.4 (*SD* = 5.9). These differences support evaluating the appropriate cutoff point according to national, not international, norms. Based on the validation of the long CAP Inventory, the cutoff point seemed to be five. According to that, six per cent of the respondents had elevated risk of child maltreatment and should be offered support for managing their everyday lives with children. From the preventive perspective, in the general population all the parents’ worry items should be taken seriously, and a family’s need for support should be discussed genuinely regardless of the abuse score.

The validity scales (the Lie Scale and the Random Response Scale) seemed to work in the Finnish sample when they were considered together (only 5.9% of invalid responses). It also seems that the measure was easy to answer, while the number of missing responses was generally low. This differs from the American and UK samples, in which the number of invalid responses varied between 27% and 31.9% [12]. It should also be considered that according to the long CAP Inventory, the cutoff scores for the Lie and Random Response scales should be set to the 95th percentile of the frequency distribution. Therefore, in the Finnish sample in the Lie Scale cutoff point would be five or more, not four or more as in the American or UK sample. Then the proportion of invalid responses would be 2.9% if the Lie Scale and the Random Response Scale were considered together.

The factor structure shows that the Abuse Risk Scale includes a range of dimensions associated with physical child abuse. The analysis mirrors to some extent the US and UK analyses although the analysis suggested five factor solution. In American sample, a seven-factor structure [15] and in the UK sample [17] a six-factor solution were considered best. Happiness, which was found in the US and UK samples, was not identified in the Finnish sample, because of the lack of variation in all items reflecting happiness. Loneliness and distress were separate factors in the US and UK samples, but they are in one factor for the Finnish sample. Rigidity and family conflict were also found in the US and UK samples. Impact of others was found in the UK sample as a factor of its own, but in the US sample, those items were connected to the family conflict factor. In the UK sample, there was no financial insecurity factor, and items reflecting financial insecurity in the US sample were connected to happiness in the UK sample. In the Finnish sample, in the fifth factor only one item was loaded: ‘I sometimes worry that I will not have enough to eat.’ Therefore, this factor could be seen as a financial insecurity factor.

### Strengths and weaknesses of the study

The size of the data (*N* = 453) was quite good and bigger than the US or UK validation analysis, but because the rate of the Abuse Risk Scale is so low, the number of respondents with elevated risk for child maltreatment is quite low (27). The response rate was 61 and the characteristics of non-respondents are not available for analysis. Although the response rate is quite good, there is no guarantee of the representativeness of the data. Almost complete lack of variation in items reflecting adversity and poor mental health (happiness items, being upset etc.) suggests that responders may be positively selected. Data is also skewed based on the gender of the respondents. (females 82%). Therefore, assessing the validity of the instrument in representative samples is a considerable challenge for future research.

The data do not include information to analyze the correct classification rate, which would also be important in validating a screening measure. This is the next phase for validating the BCAP in Finnish child and family services. In this study, the BCAP was tested with the general population. According to Milner and Crouch, the correct classification rates with the CAP have been lower than 90% in more diverse populations than known abusive parents [12]. Milner and Crouch suggested that the CAP more likely fails to detect abusive (false negatives) parents than misclassifies nonabusive comparison parents as abusive (false positives). In addition, the Abuse Risk Scale specificity should be tested further for its appropriateness in medical settings as suggested [12]. Further research in the medical context is promising because most parents accepted the BCAP well, and only a few nurses reported that the parent had considered the BCAP inappropriate and annoying.

### Findings in relation to other studies

In the Finnish general population sample, the norms of the abuse score were different from those reported for US and UK samples. This is in line with the earlier research findings suggesting that rates of child maltreatment are lower in Nordic countries compared to, for example, UK. This emphasizes that also the validation of measures to detect child maltreatment potential should be validated separately in each country. In addition, the variation in some items was smaller; in four items, almost zero, which means that those items do not separate Finnish respondents. These items reflected the happiness factor. The zero variance in the happiness items may exist because the analysis was based on a general sample, it may reflect some cultural differences or it may reflect some skewness of the data. Small (or none) variation is some items emphasizes further research to analyze the validity of the instrument in samples, of which representative nature can be assessed.

The Finnish sample conducted five sub-scales when in the US sample the best factor solution was seven factors and in the UK sample six factors. Despite this difference, the factor structure in the Finnish analysis overlapped clearly with the factors from the US and UK analyses of the BCAP. In addition, factors are not used to produce individual factors scores but to ensure the Abuse Risk scale includes a range of dimensions associated with physical child abuse risk [15]. That was shown to be ensured in the Finnish sample.

Validity scores created based on the US sample seemed to work quite well in the Finnish general population, when the Lie Scale and the Random Response scale were considered together, although in the UK sample the findings were different. The rate of invalid responses was statistically significantly lower in the Finnish sample than in the US and UK samples.

Overall, the analysis of the Finnish sample concluded the same as the US and UK analyses: The BCAP could be considered a reliable, quick, and useful clinical tool for screening potential child maltreatment among parents when the original longer version of the CAP Inventory is seen as too exhausting for practitioners. This is an important finding in progress of further evaluation of the use of the BCAP. Further research is, however, needed to analyze the correct classification rate of the measure and to validate the instrument in a sample, which representativeness can be evaluated.

### Implications

Assessment practices for the risk of child maltreatment, as well as other practices [19], need to be nationally developed, and based on scientifically and clinically tested international instruments. It is always more ethical and cost-effective to prevent child maltreatment than only treat child maltreatment injuries. Findings of the validation of the CAP Inventory in Finland [13], and clinical testing of the BCAP, this instrument could be considered as a valid instrument. The evaluation process will continue by launching an electronic version of the BCAP and its manual, and by continuation of the research especially related to correct classification rate and the role of validity scales in clinical work.

## References

[1] Durrant JE, Smith AB Global pathways to abolishing physical punishment: realizing children’s rights. New York: Routledge; 2011.

[2] Fagerlund M, Peltola M, Kääriäinen J, et al. Lasten ja nuorten väkivaltakokemukset 2013: Lapsiuhritutkimuksen tuloksia. [Experiences of violence among children and young people 2013: results from the Finnish child victim survey]. Tampere, Finland: Police University College; 2014.

[3] Ellonen N, Tindberg Y, Lucas S, et al. Parents’ self-reported use of corporal punishment and other humiliating upbringing practices in Finland and Sweden—A comparative study. Child Abuse Rev. 2017;26:289–304.

[4] Clément ME, Bouchard C Predicting the use of single versus multiple types of violence towards children in a representative sample of Quebec families. Child Abuse Neglect. 2005;29:1121–1139. 16315354 10.1016/j.chiabu.2005.04.006

[5] Radford L, Corral S, Bradley C, et al. The prevalence and impact of child maltreatment in the United Kingdom. Child Abuse Neglect. 2013;37:801–813. 23522961 10.1016/j.chiabu.2013.02.004

[6] Paavilainen E, Flinck A 2015 Efficient methods for identifying child maltreatment in social and health care. Nursing Research Foundation: Clinical guidelines – from evidence to outcomes. http://www.hotus.fi/system/files/SUOSITUS_lasten_kaltoinkohtelu_ENGLANTI.pdf

[7] Norman RE, Byambaa M, De R, et al. The long-term health consequences of child physical abuse, emotional abuse, and neglect: a systematic review and meta-analysis. PLOS Med. 2012;9:11. 10.1371/journal.pmed.1001349 PMC3507962 23209385

[8] Bailhache M, Leroy V, Pillet P, et al. Is early detection of abused children possible?: a systematic review of the diagnostic accuracy of the identification of abused children. BMC Pediatr. 2013;13:202. 24314318 10.1186/1471-2431-13-202 PMC4029314

[9] Heinze MC, Grisso T Review of instruments assessing parenting competencies used in child custody evaluations. Behav Sci Law. 1996;14:293–313.

[10] Milner J The Child Abuse Potential Inventory manual. 2nd ed Webster: Psytec; 1986.

[11] Milner JS, Crouch JL Psychometric characteristic of translated versions of the Child Abuse Potential Inventory. Psychol Violence. 2012;2:239–259.

[12] Milner JS, Crouch JL Child physical abuse risk assessment: parent and family evaluations In: Campbell JC, Messing JT, editors. Assessing dangerousness – Domestic violence offenders and child abusers. 3rd ed New York: Springer; 2017 p. 55–88.

[13] Ellonen N, Lepistö S, Helminen M, et al. Cross-cultural validation of the Child Abuse Potential Inventory in Finland: preliminary findings of the study among parents expecting a baby. J Soc Serv Res. 2017;43:308–318.

[14] Lepistö S, Ellonen N, Helminen M, et al. Family health, functioning, social support and child maltreatment risk of families expecting a baby. J Clin Nurs. 2017;26:2439–2451. 27681640 10.1111/jocn.13602

[15] Ondersma S, Caffin M, Mullins S, et al. A brief form of the Child Abuse Potential Inventory: development and validation. JCCAP. 2005;34:301–311. 10.1207/s15374424jccp3402_9 15901230

[16] Ono M, Honda S Association between social support and child abuse potential among Japanese mothers. Child Youth Serv Rev. 2017;73:88–92.

[17] Walker C, Davies J A cross-cultural validation of the Brief Child Abuse Potential Inventory (BCAP). J Fam Viol. 2012;27:697–705.

[18] Haapasalo J, Aaltonen T Child abuse potential. J Interpers Violence. 1999;14:571–585.

[19] Ahlforn KC, Horwitz EB, Osika W A Swedish version of the Consultation and Relational Empathy (CARE) measure. Scand J Primary Health Care. 2017;35:286–292. 10.1080/02813432.2017.1358853 PMC5592356 28768444

